# IL-1β induces apoptosis and autophagy via mitochondria pathway in human degenerative nucleus pulposus cells

**DOI:** 10.1038/srep41067

**Published:** 2017-01-25

**Authors:** Jieliang Shen, Shengxi Xu, Hao Zhou, Huzhe Liu, Wei Jiang, Jie Hao, Zhenming Hu

**Affiliations:** 1 Department of Orthopaedic Surgery, the First Affiliated Hospital of Chongqing Medical University, Chongqing, China

## Abstract

IL-1β has been reported highly expressed in degenerative intervertebral disc, and our previous study indicated IL-1β facilitates apoptosis of human degenerative nucleus pulposus (NP) cell. However, the underlying molecular mechanism remains unclear. We here demonstrate that IL-1β played a significantly pro-apoptotic effect under serum deprivation. IL-1β decreased Bcl-2/Bax ratio and enhanced cytochrome C released from mitochondria to cytosol, which proved mitochondria-meidated apoptosis was induced. Subsequently, mitochondria damage was detected under IL-1β stimualtion. In addition, IL-1β-mediated injuried mitochondria contributes to activate autophagy. However, pretreatment with the autophagy inhibitor 3-methyladenine showed the potential in further elevating the apoptosis rate induced by IL-1β in NP cells. Our results indicated that the mitochondrial pathway was involved in IL-1β-induced apoptosis of NP cells. Meanwhile, the damaged mitochondria-induced autophagy played a protective role against apoptosis, suggesting a postive feedback mechanism under inflammatory stress.

Intervertebral disc degeneration (IVDD) is an age-dependent molecular degenerative process, and its associated back pain generates a heavy economic burden on the aging society^1^. The inner nucleus pulposus (NP) tissue changes most during degeneration, including cell death enhancement, extracellular matrix destruction and inflammatory factors accumulation, which result in reduction of the spinal biomechanics and cause back pain^2^. Interleukin (IL)−1β is considered to be the most important cytokine involved in multiple pathological processes of IVDD^3^,^4^. Our previous work has indicated that IL-1β promotes the human degenerative NP cell apoptosis via its downstream signaling target NF-κB^5^. However, the underlying mechanism of IL-1β-induced apoptosis in degenerative NP cells remains enigmatic.

Progressive accumulation of damaged macromolecules leading to cell dysfunction and death is a major characteristic of age-related diseases^6^. Mitochondria are master subcellular organelles that produce and supply energy to maintain intracellular homeostasis. Under stressed conditions, dysregulated mitochondria release a set of molecules to activate downstream mitochondrial apoptotic pathway^7^. Recent evidence has suggested IL-1β induces excessive accumulation of ROS in bovine NP cells, which causes oxidative stress^8^. However, there is no direct evidence whether IL-1β could induce mitochondria-mediated apoptosis in human NP cells. In addition, autophagy is found to be activated by damaged mitochondria to maintian intercellular homeostasis, and regulate cellular loss against apoptosis^9^. Our previous work also confirmed that promoting autophagy could inhibit apoptosis in human NP cells^10^. Up to date, no study has concerned the role of IL-1β on the apoptosis and autophagy in degenerative NP cells. In the present study, we set out to investigate whether IL-1β induced apoptosis via mitochondria pathway, if so, whether the damaged mitochondria would further activate autophagy. We believe to clarify the apoptosis and autophagy responding to IL-1β stress is important for better understanding the mechanism of IVDD.

## Results

### IL-1β expression and cell apoptosis *in situ* detection

**Figure 1:**
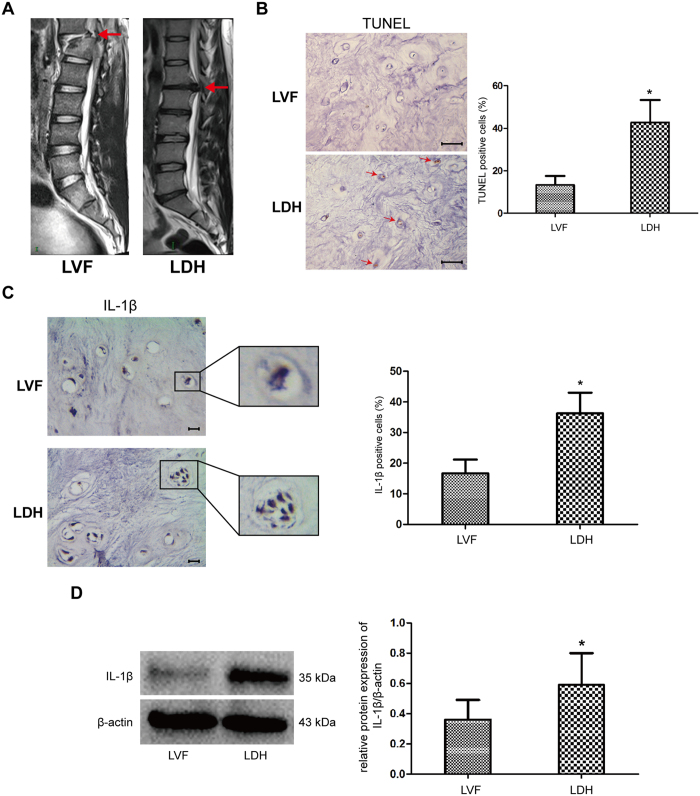
IL-1β expression is associated with cell apoptosis in NP tissues. (**A**) Representative lumbar MRI of one patient with LVF (left) and the other with LDH (right) were classified according to Pfirrmann’s grading system. The red arrows represented the grade II disc in normal group and grade IV disc in degenerative group. (**B**) *In situ* apoptotic cells were determined by TUNEL staining in NP tissues. C and D, *In situ* IL-1β protein expressions from normal and degenerative NP tissues were determined by immunological histological chemistry and western blot. *P < 0.05 Vs. LVF group. Bars = 100 μm.

First, we evaluated the relationship between IL-1β expression and apoptosis incedence in NP tissues. Representative MRI scans of patients with LVF and LDH were shown in Fig. 1A. TUNEL assay showed that the number of TUNEL positive cells was a 37.4% and 8.2% amount in the degenerative and normal group, respectively, suggesting increased cell apoptosis was demonstrated in degenerative NP tissues (Fig. 1B). Immunological histological chemistry (IHC) for IL-1β showed that cell clusters were formed within NP tissue in degenerative disc, meanwhile, IL-1β immunostaining was generally observed in the cytoplasm of NP cells in all samples. However, IL-1β showed siginificantly more immunopositive cells in the degenerative group (Fig. 1C). In parallel, western blot indicated that IL-1β protein expression was markedly higher in the degenerative NP tissues from LDH patients, compared to those from nondegenerative LVF patients (Fig. 1D).

### IL-1β induced cell apoptosis under serum deprivation

**Figure 2:**
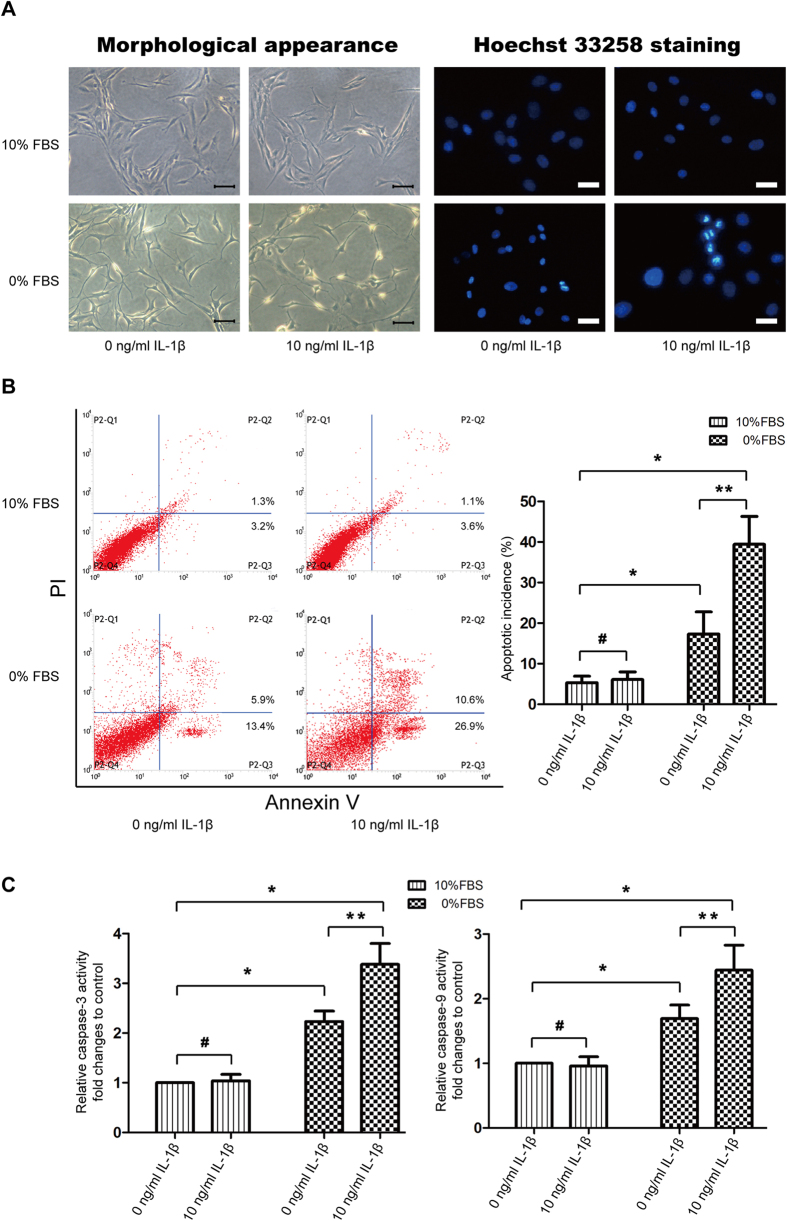
IL-1β induces cell apoptosis under serum deprivation. (**A**) Morphologic changes in apoptotic NP cells. Left series is phase-contrast photomicrograph of NP cells and right series is apoptotic nuclei brightly stained by Hoechst 33258. Amplification: 200×. (**B**) Apoptotic cells were stained with Annexin V-PE and PI, and analyzed by flow cytometry. (**C**) Caspase-3 and -9 activity were determined by special Caspase Activity Assay Kits. Relative activity of caspase-3 and -9 was represented as fold changes to control (10%FBS + 0 ng/ml IL-1β group). *P < 0.05 and ^#^P > 0.05 Vs. 10% FBS group, **P < 0.05 Vs. 0% FBS group.

IL-1β stimulation under serum-free medium led to obviously morphological changes that cells turned slender with plasma membrane blebbing, and Hoechst 33258 staining showed more apoptotic cells with high bright fluorescent nuclei. However, no significant changes were observed when NP cells were cultured under complete culture medium with 0 or 10ng/ml IL-1β (Fig. 2A). Flow cytometric analysis with Annexin-V/PI stainning indicated that serum deprivation led to a moderate increase in cell apoptosis, but IL-1β further enhanced the number of apoptotic cells (Fig. 2B). Associated with increased apoptotic incidence, colorimetric assay revealed that the activities of caspase-3 and -9 increased to ~2.2 folds and ~1.7 folds under serum deprivation, but IL-1β significantly enhanced this effect on caspase activation, correspondingly up to ~3.4 folds and ~2.4 folds, compared with control group. Il-1β in complete culture medium showed no significant effect on caspase-3 and -9 activities (Fig. 2C).

### IL-1β induced mitochondria-meidated apoptosis

**Figure 3:**
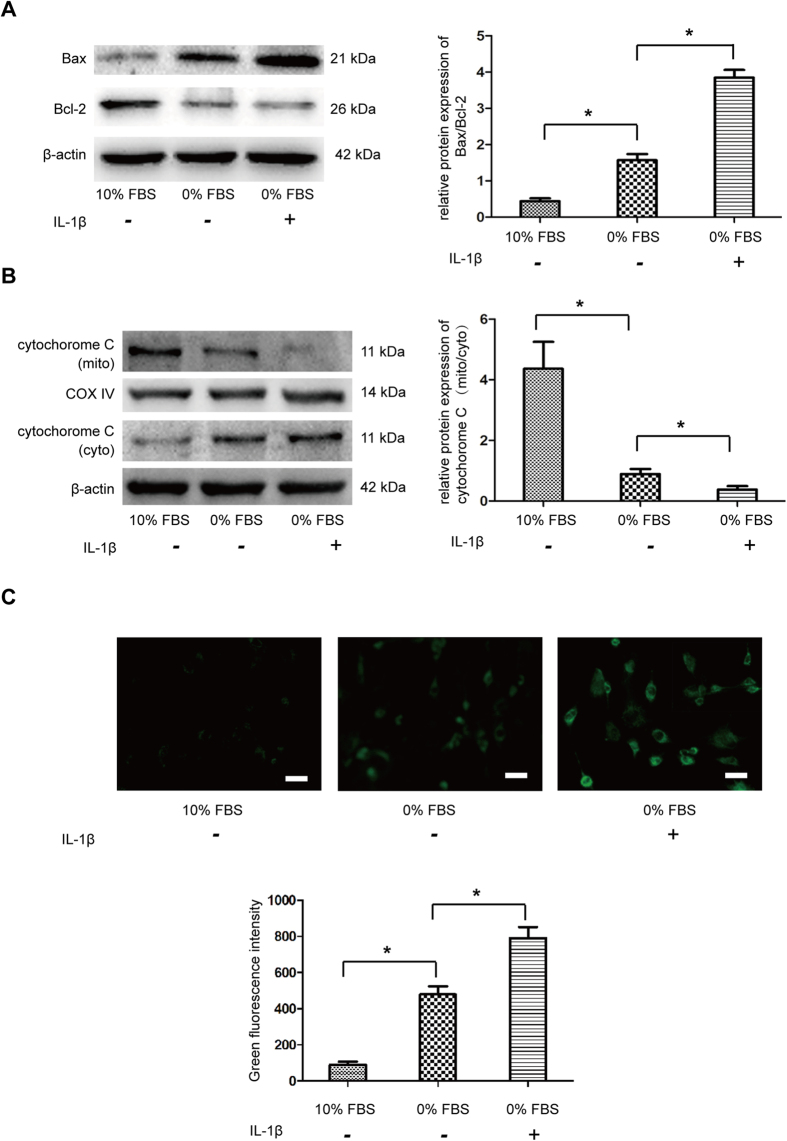
Effect of IL-1β on apoptosis mediated through the mitochodrial pathway in NP cells. (**A**) Western blot analysis for the protein expression of Bax, Bcl-2. The ratio of Bax/Bcl-2 was quantified. (**B**) Western blot analysis for the protein expression of cytochrome c from mitochondria and cytoplasm, respectively. The ratio of cytochrome c (mito)/cytochrome c (cyto) was quantified. (**C**) The intracellular ROS levels were measured by flow cytometry through DCFH-DA staining. The mean fluorescence intensity (MFI) of 10,000 cells was recorded. *P < 0.05 Vs. 0% FBS group.

Since caspase-3 and -9 were found to be activated under IL-1β treatment, the mitochondrial apoptotic pathway were first analyzed by western blot. Results showed that IL-1β significantly increased pro-apoptotic protein Bax and decreased anti-apoptotic protein Bcl-2 (Fig. 3A). Simultaneously, expression of cytochrome c from mitochondria decreased and that from cytoplasm increased under IL-1β treatment, suggesting cytochrome c was translocated from mitochondria to cytoplasm (Fig. 3B). ROS accumulation is another important mitochondrial event during apoptosis. Indeed, there is significantly increased ROS associated with IL-1β treatment compared to serum deprivation and control group (Fig. 3C). All these resutls indicated that the mitochondrial pathway was involved in the IL-1β induced apoptosis of NP cells.

### IL-1β induced mitochondria damage

**Figure 4:**
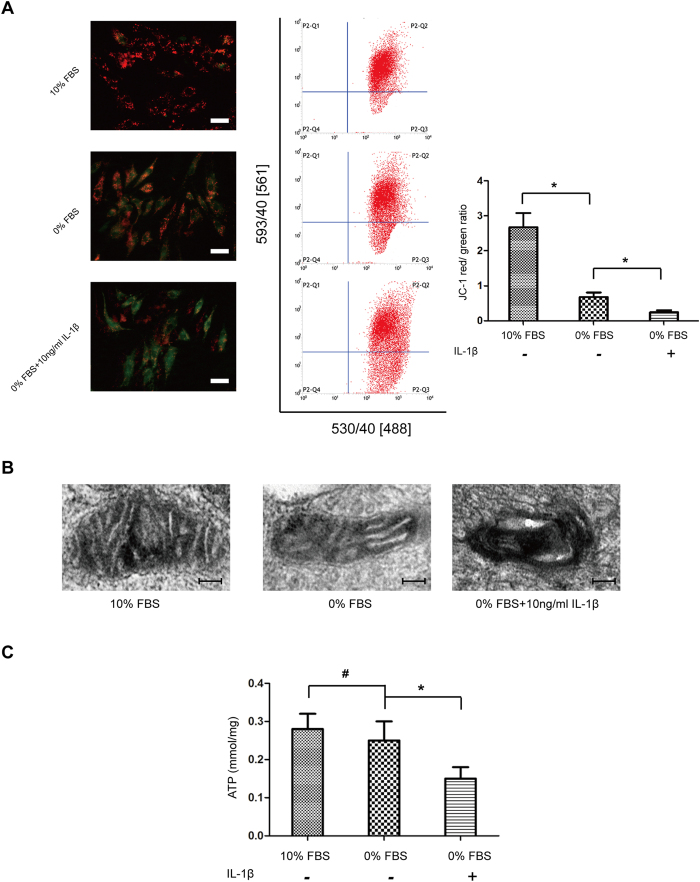
IL-1β stress leads to mitochondrial dysfunction. (**A**) The mitochondrial membrane potential was determined by staining with the mitochondrial dye JC-1. The quantitative ΔΨm was expressed as the ratio of red/green fluorescence intensity by flow cytometry. (**B**) Electron micrographs of mitochondria in NP cells treated with 10% FBS, 0% FBS and 0% FBS + 10 ng/ml IL-1β, respectively. Amplification: 2000×. (**C**) The intracellular ATP content was performed using an ATP Assay Kit by a microbeta counter. Results were normalized to cellular protein concentration for each sample. Three independent experiments were performed, and data of analysis represents the mean ± SD, *P < 0.05 and ^#^P > 0.05 Vs. 0% FBS group.

Damaged mitochondria were suggested to trigger downstream apoptotic pathway. To directly test the role of IL-1β on the mitochondria, we first measured mitochondrial membrane potential (ΔΨm). After IL-1β treatment, the red/green fluorescence ratio of NP cells significantly decreased, as observed by fluorescence microscope and flow cytometry, suggesting ΔΨm decreased by IL-1β (Fig. 4A). To confirm this finding, TEM was used to assess mitochondrial integrity and state. Serum deprivation induced a few swollen mitochondira in NP cells, but accumulation of highly damaged, electron-dense mitochondria were observed under IL-1β treatment (Fig. 4B). Energy production is the most imporant mitochondrial function, we analyzed the changes in ATP levels following IL-1β treatment. Serum deprivation alone seemed no influence on the ATP level, but exposure to IL1-β significantly decreased ATP level (Fig. 4C). All these results demonstrated that IL-1β cuased mitochondrial damage and led to cell apoptosis in degenerative NP cells.

### IL-1β stimulated autophagic flux in NP cells

**Figure 5:**
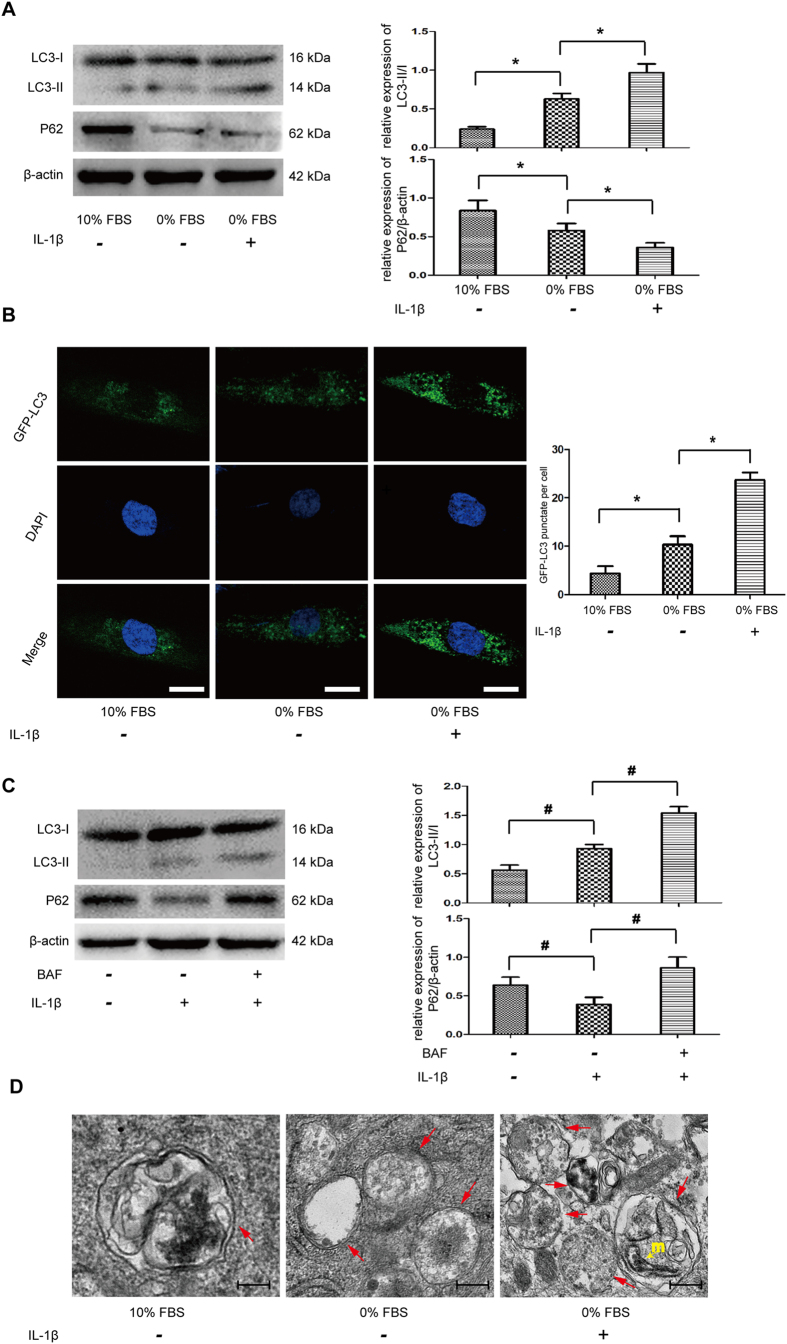
IL-1β induced autophagy in NP cells. (**A**) Western blot for the protein level of LC3 II/I and P62. (**B**) NP cells were transfected with adenovirus containing GFP-LC3. the formation and distribution of GFP-LC3 punctate were observed under confocal microscopy. Amplification: 800×. (**C**) Autophagy flux determination using Bafilomycin A1 pre-incubation with NP cells, western blot analyzed the exprssions of LC3 II/I and P62. (**D**) Morphological observation of autophagy under transmission electron microscope. Red arrows represent the characteristic double-membrane ultrastructural morphology of autophagic vacuoles. In the 0% FBS + 10 ng/ml IL-1β group, we observed double-membrane vesicles formed around pieces of mitochondrion (yellow arrow). *P < 0.05 Vs. 0% FBS group, ^#^P < 0.05 Vs. IL-1β group.

Autophagy is found to be triggered by mitochondrial damage, which is a quality control process to mediate selective injuried mitochondira removal. To determine the activation of autophagy under mitochondrial dysfunction conditions, western blot showed that IL-1β significantly changed the autophagic marker LC-3II and P62/SQSTM1 expressions (Fig. 5A). Fluorescence-based detection of LC-3 isoforms were directly observed in NP cells, IL-1β treatment resulted in a significant improvement in the LC3 puncta, indicative of increased autophagosome formation (Fig. 5B). These results indicated that IL-1β actually up-regulated the autophagy activity in NP cells. To assess this autophagy flux, we treated cells with a lysosomal inhibitor, bafilomycin A1. The addition of bafilomycin A1 further increased LC3-II and p62/SQSTM1 accumulation, compared with cells treated with IL-1β only, which indicated IL-1β-mediated autophagy is not because of reduced autophagosome turnover, but increased autophagic flux (Fig. 5C). In addition, TEM observation found typical double-membraned autophagosomes in NP cells from serum deprivation group. Moreover, mitochondrial fragmentations were observed and sections of the mitochondrion appeared to be surrounded by double-membrane profiles under IL-1β treatment (Fig. 5D).

### IL-1β-meidiated autophagy played pro-survival function

**Figure 6:**
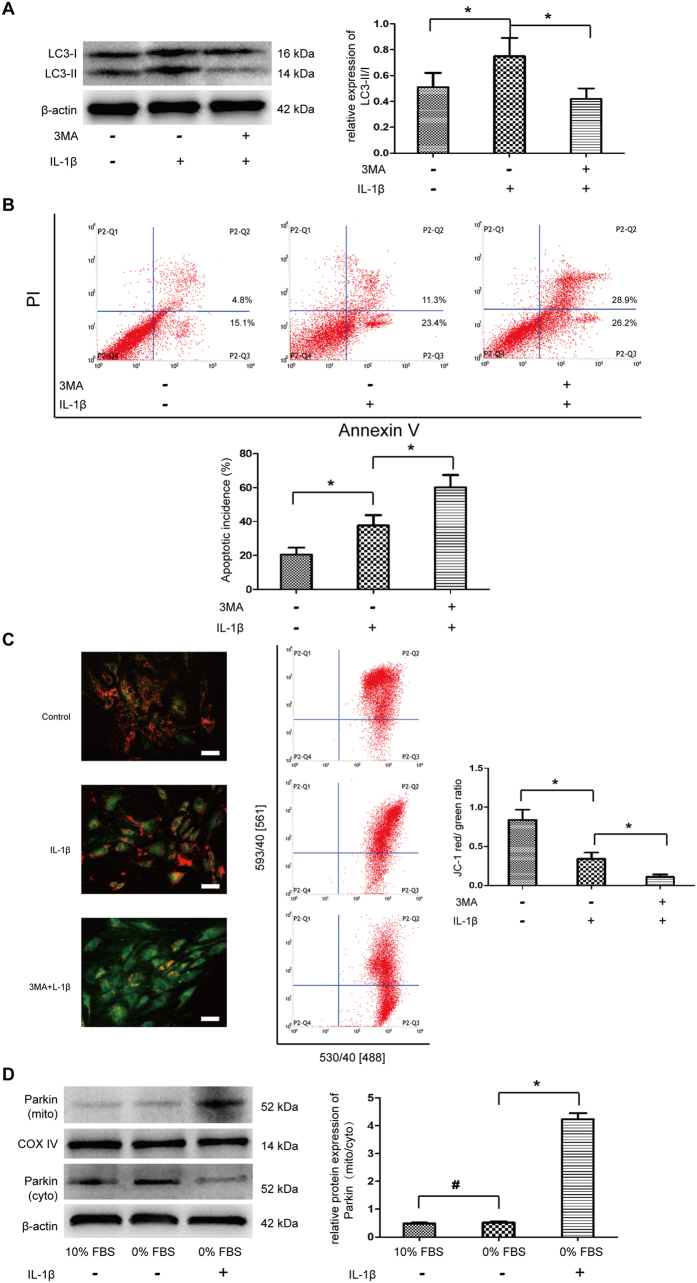
The interactions between apoptosis and autophagy in NP cells under IL-1β stress. NP cells were pre-treated 3MA for 2 h and then stimulated with IL-1β under serum deprivation. (**A**) Western blot analysis for LC II/I. (**B**) Apoptosis incidence quantified by flow cytometry by Annexin V and PI staining. (**C**) The mitochondria membrane potential was measured by flow cytometry through JC-1 staining. (**D**) Western blot analysis for the protein expression of Parkin in cytoplasm and mitochondria, respectively. The ratio of Parkin (mito)/Parkin (cyto) was quantified. *P < 0.05 Vs. IL-1β group, and ^#^P > 0.05 Vs. 10% FBS group.

To evaluate the role of IL-1β-mediated autophagy on the cell apoptosis, NP cells in serum deprived medium were pre-treated with 3MA to inhibit the autophagy induction. 3MA treatment significantly attenuated the LC3-II expression that indicated 3MA decreased the autophagy incidence by IL-1β treatment (Fig. 6A). Flow cytometric analysis revealed that obviously increased apoptotic ratio was observed under 3MA treatment (Fig. 6B). The result suggested the autophagy activation induced by IL-1β may be a self-protective mechanism in NP cells. Moreover, decreased ΔΨm were confirmed following autophagy inhibition by 3MA (Fig. 6C). The selective degradation of mitochondria by autophagy is often based on Parkin-depend manner^11^. Western blot analysis showed that serum starvation had little effect on the translocation of Parkin, but IL-1β recruited Parkin from cytoplasm onto depolarized mitochondria (Fig. 6D), which indicated that IL-1β-meidiated autophagy may be a critical homeostasis mechanism, as mitophagy.

## Discussion

The results of this study demonstrate for the first time that IL-1β facilitates apoptosis through mitochondrial apoptotic pathway in human degenerative NP cells. In turn, the accumulation of damaged mitochondria enhances autophagy activation, which plays a pro-survival role against apoptosis. These findings suggest there existing a functional loop between autophagy, apoptosis and IL-1β in NP cells, of which mitochondria seems to be a key pivot.

In this study, IHC results clearly showed that in addition to immune cells, NP cells themselves are able to produce IL-1β. Furthermore, cell apoptotic rate and IL-1β expression were found to be more prominent in the dengerative NP tissues. In consideration of nutrition deficiency for NP cells during IVDD^12^, and our finding that appearance of cell clusters in degenerative NP tissues, these results suggested that degenerative NP cells settle in a harsh microenvironment with local high concentration of IL-1β and extremely low nutrition supply. *In vitro*, IL-1β was found to facilitate NP cell apoptosis under serum deprivation. Interestingly, IL-1β showed no significant influence on cell apoptosis in the presence of serum supply, suggesting nutritionally adequate cells were not sensitive to inflammatory stress. Zhao *et al*. reported a similar finding on rat annular cell apoptosis^13^. This apoptotic model induced by IL-1β mimics that the NP cells suffers oligotrophic condition under IL-1β stress *in vivo*.

Mitochondrial pathway is considered to be involved in compression-induced apoptosis of rabbit NP cells^14^. Some circumstantial evidence have suggested IL-1β could aggravate oxidative stress in NP cells. Yang *et al*. reported that glutathione could protect human NP cells from IL-1β-induced apoptosis, which is a powerful antioxidant in human cytoplasm^15^. Intracellular oxidative stress is always inducd by ROS accumulation mediated by mitochondria damage^16^, thus we speculated IL-1β induced apoptosis via mitochondrial pathway. Up to date, our study is the first report to directly demonstrate that IL-1β induced mitochondrial pathway in NP cells, by increasing expressoin ratio of Bax/Bcl-2, releasing cytochrome c from mitochondria to cytoplasm, subsequently activating downstream caspase-9 and caspase-3 to complete the apoptosis process. Mitochondria are important for regulating cell survival, and cell apoptosis is always triggered by dysfunctional mitochondria^17^. Under conditions where IL-1β induced mitochondria-depend apoptosis in NP cells, mitochondrial damage in forms of depolarization, loss of ATP production, and increasing ROS level were detected in this study. Taking together, we provide evidence indicating that IL-1β induces mitochondrial damage, leads to apoptotsis through mitochondrial apoptotic pathway in NP cells.

Autophagy is a cellular degradation pathway essential for survival, our previous work has showed that activated autophagy protects against apoptosis in NP cells. Damaged mitochondria served as the basic mechanism mediating cell apoptosis and autophagy^18^, which would result in mitochondria-depend apoptosis, meanwile induce autophagy to remove dysfuncional mitochondria to maintain cellular homeostasis. The datas of the present study found an early activation of autophagy at 24 hours in response to mitochondrial dysfunction injuried by IL-1β. Thus, we proved that IL-1β could induce apoptosis and autophagy in NP cells, but the relationships between them were still unclear. According to the available evidence, the role of autophagy on the survival of NP cells is a double-edged sword by adjusting to variational stress^19^. In this study, 3MA pretreatment dramatically further increased IL-1β-induced apoptosis, revealing that autophagy activation may protect against cell apoptosis as a compensatory mechanism.

Damaged mitochondria can be degraded by a selective type of autophagy, which is termed mitophagy. The physiological significance of mitophagy is timely elimination of damaged and dysfunctional mitochodnria in preventing from the release of pro-apoptotic proteins which causes apoptosis and inflammasome activation^20^. However, to our knowledge, the phenomenon of mitophagy in dengenerative human NP cells has never been reported. Our study preliminary showed that the autophagy inhibition by 3MA further decreased the mitochondrial membrane potential, which suggeste that the autophagy induced by IL-1β was partially involved in mitochondria control. Parkin is an E3 ubiquitin ligase that has been reported to initiate mitophagy^21^, western blot analysis directly indicated that IL-β promoted translocation of Parkin to the mitochondria, suggesting the involvement of Parkin-mediated mitophagy in NP cells was under autophagy activation. Further evidence about mitophagy was revealed by electron microscopy that some mitochondria-like subcellular fraction were incorporated into autophagic vacuoles under IL-1β treatment. On the basis of these results, we think IL-1β induced mitochondria damage was at least partially promote mitophagy activation, which plays an anti-apoptotic role as a compensatory mechanism under inflammatory stress.

In conclusion, our study found that mitochondrial apoptosis pathway was involved in IL-1β-induced apoptosis in NP cells, autophagy triggered by IL-1β in turn played a pro-survival role. However, defective autophagy in the degenerative NP cells, as we previous reported^10^, is not sufficient to maitain cell balance, ultimately the presence of inflammatory stress results in cell death. These results will enrich our understanding of the mechanisms mediating IL-1β-induced NP cell death and the relationships between autophagy and apoptosis under IL-1β stress.Pharmacologic augmentation of autophagy seems a potential treatment for IVDD.

## Materials and Methods

### NP sample source

We prospectively enrolled and collected 7 patients with lumbar disc hernia (LDH) suffering from sciatic and back pain as degenerative group, and 5 age-matched patients with lumbar vertebral fracture (LVF) as normal control, who have undergone discectomy in our department. Degenerative grades of the IVDs were according to the Pfirrmann classification by pre-operative MRI scans^22^. We defined the normal disc according to the Pfirrmann grade I or II, and the tissue donors without documented medical history of low back pain. Details for human nucleus pulposus samples processing were seen in Supplementary Table 1.

Written informed consent was obtained from all tissue donors and this study was approved by Ethics Committee of Chongqing Medical University before surgery. All samples were obtained in accordance with the World Medical Association Declaration of Helsinki Ethical Principles for Medical Research Involving Human Subjects.

### *In situ* TUNEL and IL-1β staining

*In situ* analysis of apoptotic cells and IL-1β production in NP tissues were performed using a TUNEL assay kit (Roche, Germany) and a streptavidin–peroxidase IHC kit (Boster, China), respectively. NP tissues from degenerative and normal groups were embedded in paraffin and cut serially into 5 μm-thick sections for respective staining, which were applied to the sections according to the manufacturer’s instructions. The color was developed with DAB and hematoxylin was used as a counter stain. The positive stained cells were counted in 3 different areas under microscope.

### NP cell isolation and culture

NP tissues from LDH donors were isolated and cultured as described previously^23^. In brief, NP samples were first separated from harvest IVD tissues microscopically according to their morphological difference; Then, after washing by PBS solution, samples were sequentially digested by 0.25% trypsin solution and 0.2% type II collagenase (sigma, USA) at 37 °C for 3~5 h; Tissue debris was removed by a 200-μm filter and the NP cells were resuspended in DMEM/F12 medium (Gibco, USA) containing 10% FBS (Corning, USA). The primary NP cells were monolayer cultured in a humidified atmosphere containing 5% CO_2_ at 37 °C. Third-passage cells were used throughout this study.

### Cell treatment

In order to synchronize the cells and reduce the effect of starvation on autophagic level, cells were cultured with DMEM/F-12 medium with 2% FBS for 12 h before treatment and was changed by complete culture medium with 10% FBS or serum-free medium with 10 ng/ml IL-1β for another 24 h. The concentration of IL-1β used in this study was determined by the concentration gradient experiment, details were seen in Supplementary Figure 1. In addition, Bafilomycin A1 (0.1 μM) was used to analyze the autophagic flux and 3-methyladenine (5 mM) was used to inhibit autophagy, which were both applied 2 h prior medium change.

### Morphologic observation of the apoptotic cells

NP cells were prepared at a density of 1 × 10^5^/well in a 6-well plate. After various treatment, cells were fixed with 4% paraformaldehyde for 15 min, then observed under an inverted microscope. To further detect the apoptotic features, Hoechst 33258 was used to stain the morphologic changes in apoptotic nuclei. Staining with 2 μg/ml Hoechst 33258 (Beyotime, China) solution for 5 min, apoptotic cells from normal ones were discriminated by showing brightly fluorescent DNA fragmentation and chromatin condensation under fluorescence microscopy.

### Apoptotic incidence detection by Annexin V-FITC/PI

Apoptotic incidence was detected by flow cytometry with Annexin V-PE/PI apoptosis detection kit (BD Biosciences, USA) as previously described^24^. In briefly, after treatment, cells were collected and suspended in 1× Binding buffer, and 1 × 10^5^ cells were counted and incubated with 5 μl of Annexin V-PE and 5 μl of PI for 30 min at room temperature in the dark. Then 1× Ending buffer was added and flow cytometry analysis was performed within 1 h. Apoptotic cells were stained as Annexin V (+)/PI (−) and Annexin V (+)/PI (+).

### Caspase activity measurement

Caspase activities were measured as the protocol provided by Caspase-3 And -9 Activity Assay Kit (Beyotime, China). Briefly, cell lysates were harvested, and the synthetic colorimetric substrates were added to equal amounts of proteins from each sample at 37 °C for 2 h, followed by reading the samples at 405 nm on the microplate reader.

### Western blot

Tissues and whole cell were lysed on ice using RIPA Lysis Buffer (Beyotime, China) or Mitochondrial and Cytosolic Fractions Isolation Kit (KeyGEN BioTECH, China) according to the manufacturer’s instructions. After protein transfer, the membranes were blocked by Blocking buffer (Abcam, UK) for 1 h at 37 °C and then incubated with primary anti-IL-1β (Santa Cruz, USA), Bcl-2, Bax, Cytochrome C (Cell Signaling, USA), β-atcin (Beyotime, China), LC3B, P62/SQSTM1 and Parkin (Sigma, USA) at 4 °C overnight. After washing by Tris Buffered Saline with Tween (TBST), membranes were incubated with the respective secondary antibodies for 1 h at 37 °C. Finally, the bands were visualized by an ECL detection kit (Millipore, USA). The protein expression levels were detected by Quantity One software (Bio-Rad, USA).

### Reactive oxygen species (ROS) levels measurement

The intracellular ROS level was measured by 2′,7′-dichlorofluorescin diacetate (DCFH-DA; Beyotime, China), which is oxidized into the fluorescent dichlorofluorescein (DCF) in the presence of ROS. Briefly, 1 × 10^4^ colleceted NP cells were resuspended in DCFH-DA and incubated in the dark at 37 °C for 30 min. Then the cells were washed and analyzed by flow cytometry with an excitation wavelength of 488 nm and an emission wavelength of 525 nm. The mean fluorescence intensity (MFI) of 10,000 cells was recorded.

### Mitochondrial membrane potentials assay

Mitochondrial membrane potential (ΔΨm) was assessed using JC-1 (Beyotime, China). Briefly, NP cells were seeded at a density of 1 × 10^4^/well in 6-well plate overnight, and after various treatment, cells were washed in PBS buffer and incubated with JC-1 fluoroprobe (2.5 μg/ml) for 30 min at 37 °C in the dark and rinsed twice with incubation buffer. Stained cells were visualized under a fluorescence microscope. Depolarized ΔΨm results in a decrease of red fluorescence and an increase of green fluorescence. The ratio of red and green fluorescence intensities were determined by flow cytometry.

### ATP levels measurement

Intracellular ATP content was detected using an ATP assay kit (Beyotime, China). Briefly, whole-cell extracts from indicated cells were lysed by somatic cellular ATP releasing reagent. After mixing with ATP detection solution containing luciferase, the bioluminescence were measured by Synergy HT luminescence plate reader. A fresh standard curve was prepared each time and ATP content was estimated according to the curve. Results were normalized to cellular protein concentration, which was determined by an Enhanced BCA Protein Assay kit (Beyotime, China).

### Confocal microscopic analysis

To visualize the autophagosome, cells were transfected with adenovirus containin GFP-LC3 (HanBio, China). Briefly, NP cells were incubated in complete medium with the adenovirus at a MOI of 50 for 6 h, then replace fresh medium for another 24 h. The transfected cells were used for subsequent experiments. Autophagy was evaluated by analyzing the number of green fluorescent puncta of autophagosomes under laser confocal microscopy.

### Transmission electron microscopy

Transmission electron microscopy (TEM) was performed using a protocol previously described by our group^10^. Briefly, cells were harvested and fixed in 2.5% glutaraldehyde, and followed by a conventional sample preparation process. Finally, the routin ultrathin sections (60 nm) were observed by a TEM (Hitachi-7500, Japan).

### Statistical analysis

All measurements were performed in triplicate. Results were expressed as mean ± standard deviation (SD). Differences between groups were analyzed by one-way analysis of variance (ANOVA) with Tukey’s post test for multiple groups using SPSS version 19.0 statistical software program (SPSS, Inc., USA). P < 0.05 was considered as statistical significance.

## Additional Information

**How to cite this article**: Shen, J. *et al*. IL-1β induces apoptosis and autophagy via mitochondria pathway in human degenerative nucleus pulposus cells. *Sci. Rep.* **7**, 41067; doi: 10.1038/srep41067 (2017).

**Publisher's note:** Springer Nature remains neutral with regard to jurisdictional claims in published maps and institutional affiliations.

## Supplementary Material

supplementary Material
