# Supplementary material for: IL-1β induces apoptosis and autophagy via mitochondria pathway in human degenerative nucleus pulposus cells
Source: Sci Rep. 2017 Jan 25;7:41067. doi: 10.1038/srep41067 (PMC5264394; doi:10.1038/srep41067)
Supplement: supplementary Material [file srep41067-s1.pdf]

IL-1β induces apoptosis and autophagy via mitochondria pathway in human degenerative nucleus pulposus cells

Jieliang Shen PhD, Shengxi Xu MS, Hao Zhou MS, Huzhe Liu MS, Wei Jiang PhD, Jie Hao MD, Zhenming Hu PhD&MD*

Department of Orthopaedic Surgery, the First Affiliated Hospital of Chongqing Medical University, Chongqing, China.

*Address correspondence to Zhenming Hu, Department of Orthopaedic Surgery, the First Affiliated Hospital of Chongqing Medical University, 1 Youyi Rd., Chongqing, 400016, China. Email: spinecenter_hu@126.com.

Table 1. Supplementary information for human nucleus pulposus sampling

LVF	Age	Gender	Disc level investigate	Degree of degeneration	Application
1	36	F	L2/3、L3/4	Pfirrmann I-II	Tissue research for histological staining and western blot
2	37	M	L1/2	Pfirrmann I	Tissue research for histological staining
3	41	M	L1/2	Pfirrmann II	Tissue research for histological staining
4	33	F	T12/L1、L1/2	Pfirrmann I-II	Tissue research for western blot
5	45	M	L3/4	Pfirrmann II	Tissue research for western blot
**LDH**	**Age**	**Gender**	**Disc level investigate**	**Degree of degeneration**	**Application**
1	42	F	L4/5、L5/S1	Pfirrmann IV	Tissue research for histological staining and western blot
2	44	M	L5/S1	Pfirrmann V	Tissue research for western blot and cell research
3	36	M	L4/5	Pfirrmann IV	In situ histological staining and cell research
4	35	F	L3/4、L5/S1	Pfirrmann IV	Tissue research for histological staining and cell research
5	51	F	L4/5	Pfirrmann V	Cell research
6	42	M	L2/3	Pfirrmann IV	Tissue research for histological staining and cell research
7	38	M	L4/5	Pfirrmann IV	Cell research

Figure 1 Concentration gradient experiment for IL-1β

Figure 1A. CCK-8 assay for the cell viability. NP cells were treated with different concentrations of IL-1β for 24 hours.

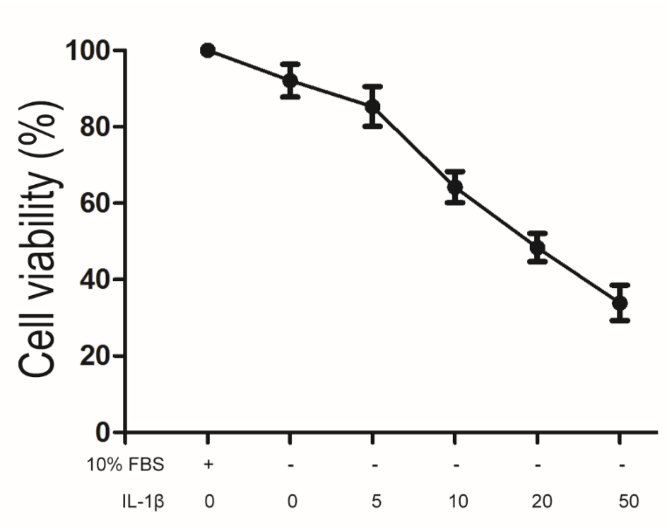

Figure 1B. Flow cytometric analysis for apoptosis incidence. The apoptosis incidence was calculated by the percentage of early apoptotic (Annexin V+/PI-) cells plus the percentage of late apoptotic (Annexin V+/ PI+) cells.

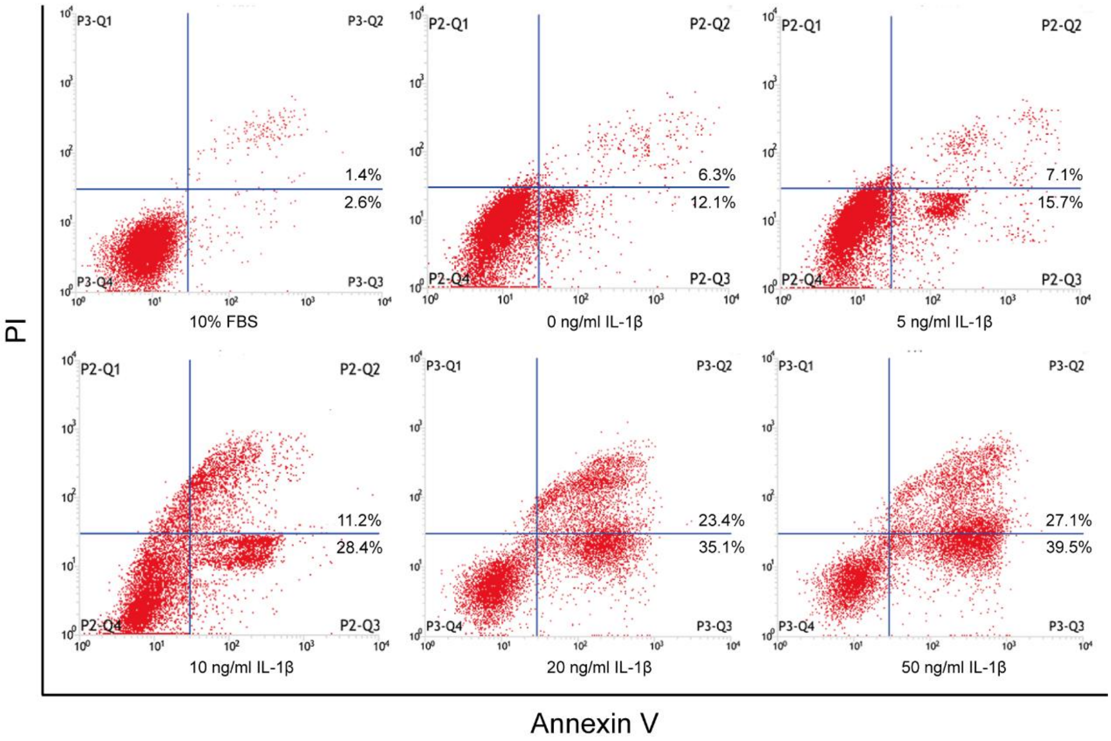

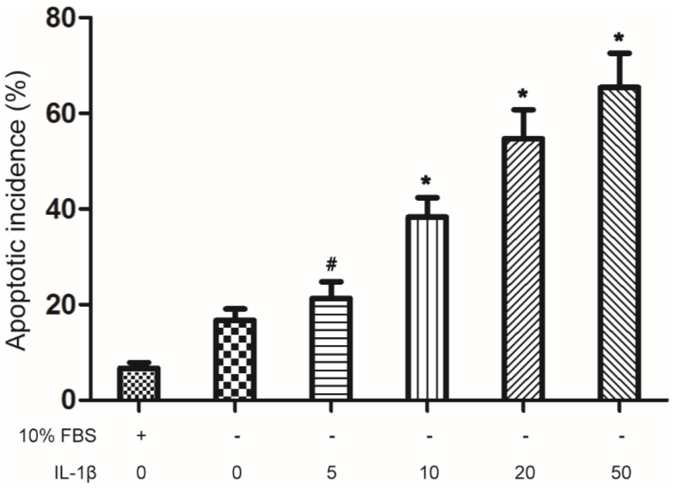

#P＞0.05 Vs. 0 ng/ml IL-1β, and *P＜0.05 Vs. 5 ng/ml IL-1β

Figure Legends

Supplementary Fiugre 1A showed CCK-8 assay for the cell viability. Results showed that the viable cells reduced to 92.1%, 85.3%, 64.2% , 48.4% and 33.9% respectively, exposing with different concentrations of 0, 5, 10, 20, 50 ng/ml IL-1β. Supplementary Fiugre 1B showed flow cytometric analysis for apoptotic incidence. Results indicated that 5 ng/ml IL-1β treatment slightly increase the apoptotic rate compared with that in 0 ng/ml group (from 16.7% to 21.3%), but 10 ng/ml IL-1β treatment significantly increased the apoptotic rate upto 38.3%. With the further increased concentration, apoptotic rate increased as high as 54.7% and 65.4% under 20 and 50 ng/ml treatment respectively. All these results suggested that 10 ng/ml IL-1β with serum-free had a definite cytotoxicity, and higher concentrations resulted in insufficient cell viablilty and too much apoptosis , thus we used the concentration of 10 ng/ml in this study.
